# Understanding and overcoming antibiotic resistance

**DOI:** 10.1371/journal.pbio.2003775

**Published:** 2017-08-23

**Authors:** Lauren A. Richardson

**Affiliations:** Public Library of Science, San Francisco, California, United States of America

Antibiotic drugs have revolutionized medicine and made our modern way of life possible. In addition to their essential role in the clinic, antibiotics are used in a huge array of non-medical applications, from promoting growth in livestock, to preserving building materials from contamination, to treating blight in orchards. However, overuse threatens their efficacy due to the promotion and spread of antibiotic resistant bacteria.

Antibiotics target and inhibit essential cellular processes, retarding growth and causing cell death. However, if bacteria are exposed to drugs below the dose required to kill all bacteria in a population (the minimum bactericidal concentration or MBC), they can mutate and resist antibiotic treatment via natural selection for resistance-conferring mutations. These genetic mutations can arise from the adoption of a plasmid encoding a resistance gene or by mutation to the bacterial chromosome itself.

The concern around the increasing prevalence of drug resistant bacteria is compounded by the fact that the discovery of new antibiotics is a fleeting rare event. Most classes of antibiotics on the market were discovered in the mid-to-late 20^th^ century. Thus, there is a limited arsenal of drugs to fight resistant bacteria, and bacteria can be resistant to multiple drugs at a time.

Given the importance of antibiotics to modern medicine, and the growing apprehension surrounding the threat of resistance, scientists are studying every aspect of antibiotic resistance. This Open Highlight features some of the cutting-edge research from the Open Access corpus on three major areas of focus: the cellular mechanisms of resistance, the evolution and spread of resistance, and techniques for combating resistance.

## Mechanisms of Resistance

A common mechanism used by bacteria to minimize the effects of antibiotics is to acquire or increase the expression of drug efflux pumps. As the name implies, these pumps expel drugs from the cytoplasm, limiting their ability to access their target. In a *PLOS Pathogens* article, researchers investigated how efflux pump expression is regulated in the human pathogen *Pseudomonas aeruginosa* [1]. They found that the multifaceted transcription regulator CpxR regulates the expression of the major efflux pump in *P*. *aeruginosa*, and is involved in modulating resistance in clinical isolates.

Resistance-conferring mutations can be specific to a particular antibiotic or they can provide protection to multiple—often related—drugs. A recent *eLife* article described a surprising set of mutations in the genes encoding components of the ribosome of *Mycobacterium smegmatis* that confer resistance to numerous antibiotics that are not related structurally or mechanistically [2]. The authors find that these mutations cause extensive changes in the transcriptome and proteome of the bacterium, including alterations to several proteins known to impact resistance. Importantly, these mutations promote further evolution of the bacteria in a multi-drug environment in a drug-specific manner, thus they both provide resistance and spur further development of resistance.

**Fig 1:**
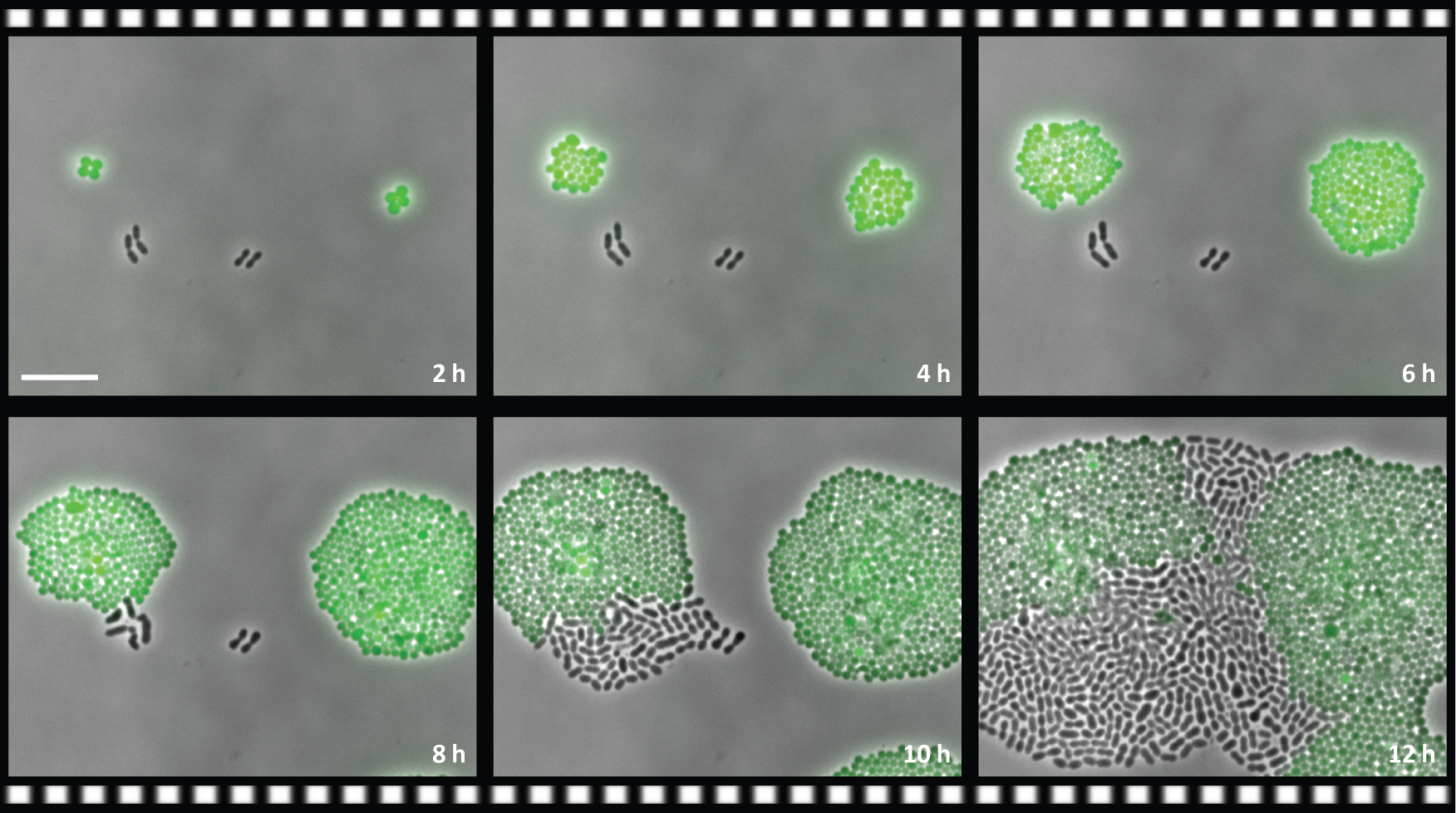
Antibiotic resistance in a microbial community. Still images of a time-lapse experiment show that the green-labeled drug-resistant strain of *Staphylococcus aureus* provides resistance for the non-labeled drug-sensitive strain of *Streptococcus pneumoniae* during antibiotic treatment. Image credit: doi: 10.1371/journal.pbio.2000631.

Resistance is not just a property of an individual bacteria, resistance can also be a property of the microbial community. In a *PLOS Biology* article, scientists show that intracellular expression of an antibiotic-metabolizing enzyme in a non-pathogenic strain of bacteria can provide resistance to the pathogen *Streptococcus pneumoniae* when the two types of bacteria are grown together, both *in vitro* and *in vivo* [3]. The expression of the metabolizing enzyme in the non-pathogenic strain deactivates the drug in the immediate environment, despite its intracellular localization, allowing for the outgrowth of the drug-sensitive pathogenic strain (Fig 1). This work highlights the importance of considering the microbial context during infectious disease.

## Evolution and spread of resistance

Since antibiotic resistance is the result of natural selection for resistance-conferring mutations, it is important to understand the evolutionary processes underlying this selection. One interesting element to this puzzle is that bacteria acquire resistance to different antibiotics at different rates. In a *PLOS Biology* article, the authors sought to understand the properties that determine how quickly resistance will evolve [4]. They identified two properties, resistance variability and dose sensitivity, that could predict the rate of evolution in seven of eight of the drugs.

Another critical element for understanding the evolution of resistance is the cost that resistance-conferring mutations have on bacterial fitness (i.e. growth rate). Most mutations have an associated cost, however bacteria can gain additional mutations, known as compensatory mutations, that offset those costs and help maintain resistance mutations in a population. A *PLOS Biology* paper describes how multi-drug resistant bacteria compensate, finding that their compensatory evolution is distinct from that of bacteria resistant to single drugs alone, due to the interaction between the resistance mutations [5].

Resistance to antibiotics is often acquired by the transfer of resistance-conferring genes between bacteria, and this acquisition is usually facilitated by a conjugative plasmid. These plasmids encode the genes necessary for two bacteria to pass the plasmid between them, and they can also encode resistance genes. But, as mentioned above, resistance comes at a cost, and a study published in *Science Reports* of the compensatory evolution of a large conjugative resistance plasmid showed that evolution follows common paths leading to plasmid stabilization and persistence of resistance [6].

**Fig 2:**
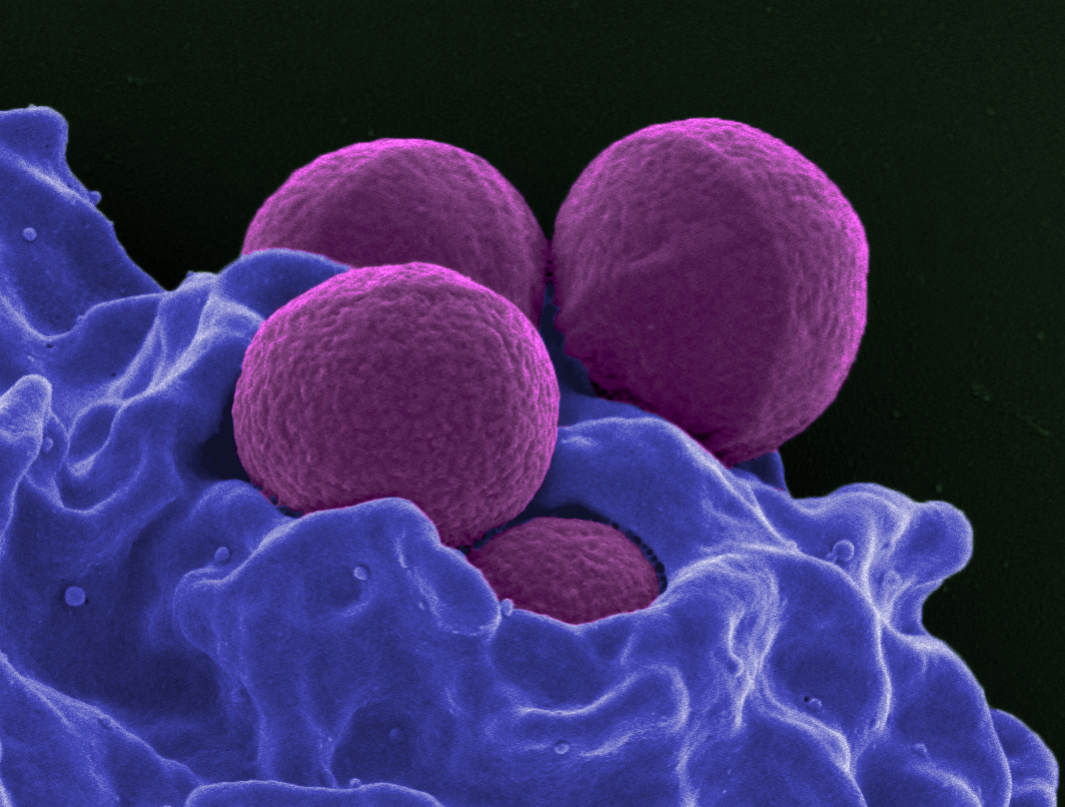
Methicillin-Resistant *Staphylococcus aureus* (MRSA). Scanning electron micrograph of a human neutrophil ingesting MRSA (purple). *Image credit*: *NIAID*

Methicillin-resistant *Staphylococcus aureus* (MRSA) is the most common antibiotic resistant infection in humans, and the most frequent mechanism of resistance in MRSA is via the acquisition of *mecA* (Fig 2). mecA is a member of the penicillin-binding protein family that doesn’t bind β-lactams (like penicillin) effectively and is thus immune to its effects. In a *PLOS Genetics* article, the scientists map the evolution of *mecA* from its original role cell wall biosynthesis [7]. They identify four mechanisms that have led to its new role in resistance, and most importantly, show that it was the use of antibiotics in medicine and in livestock feed that drove this evolution and spread of resistance.

## Techniques for combating resistance

While we only have a limited set of antibiotics in our arsenal, there are better ways of dosing and combining drugs to increase efficacy and decrease resistance. One method is using a sequential regimen. Sequential regimens alternate the use of two (or more) drugs over time. The authors of a *PLOS Biology* article showed that they could design sequential regimens that eliminated bacteria at doses that would normally lead to resistance and treatment failure [8]. The key goal is to maximize collateral sensitivity–or when one drug sensitizes the bacteria to the second drug–while minimizing cross-resistance–where resistance to one drug confers resistance to the second drug.

A pair of synergistic antibiotics are more effective than the sum of the efficacies of each antibiotic when used alone and their dual action is thought to be more difficult overcome. Unfortunately, finding synergistic pairs is difficult and traditionally requires screening a huge number of drug combinations. In a *PLOS Biology* article, researchers utilized a technique originally developed for identifying novel antifungals [9]. The method uses previously generated chemical-genetic datasets and as a proof-of-concept, the researchers identified new synergistic combinations, including one with the classic antiviral, AZT.

Even in single-drug regimens, proper dosing is essential to minimize the development of resistance. Yet, finding the optimal dosing regimen is tricky and requires costly *in vivo* experiments. By modeling and understanding the kinetics of how the drug and target interaction, an article appearing in *PLOS Computational Biology* demonstrates that the best time and concentration parameters for an antibiotic dose can be predicted [10].

A critically important but often overlooked aspect of the treatment of antibiotic-resistant infections is the role of the immune system in clearance. In an article published in *PLOS Computational Biology*, the authors use mathematical modelling of within-host infection dynamics to understand the interaction between the host immune response and antibiotic treatment [11]. By comparing a standard fixed dose and duration treatment regime to dynamic regimes that account for pathogen load, they are able to identify treatments that promote synergy between the immune system and the antibiotics.

What if, instead of treating resistant bacteria with more drugs, we could instead make them sensitive to the original drug again? That is the question asked by the authors of a recent *PLOS Biology* article [12]. They show that resistant bacteria can be re-sensitized by treating with a specifically designed anti-sense oligonucleotide. This oligonucleotide, a peptide-conjugated phosphorodiamidate morpholino oligomer (PPMO), acts as an antisense mRNA translation inhibitor, and can be designed to target the mRNAs encoding resistance genes. They found that the most effective PPMOs target a constituent of the major drug efflux pump, and treatment with this PPMO can lead to a 2- to 40-fold increase in antibiotic efficacy.

For more detailed reading please see the associated PLOS Collection [13].
